# Antiretroviral Prescribing Practices Among Pregnant Women Living With HIV in the United States, 2008-2017

**DOI:** 10.1001/jamanetworkopen.2019.17669

**Published:** 2019-12-18

**Authors:** Kathleen M. Powis, Yanling Huo, Paige L. Williams, Deborah Kacanek, Jennifer Jao, Kunjal Patel, George R. Seage, Russell B. Van Dyke, Ellen G. Chadwick

**Affiliations:** 1 Department of Internal Medicine, Massachusetts General Hospital, Boston; 2 Department of Pediatrics, Massachusetts General Hospital, Boston; 3 Department of Immunology and Infectious Diseases, Harvard T.H. Chan School of Public Health, Boston, Massachusetts; 4 Center for Biostatistics in AIDS Research, Harvard T.H. Chan School of Public Health, Boston, Massachusetts; 5 Department of Epidemiology, Harvard T.H. Chan School of Public Health, Boston, Massachusetts; 6 Department of Biostatistics, Harvard T.H. Chan School of Public Health, Boston, Massachusetts; 7 Department of Pediatrics, Feinberg School of Medicine, Northwestern University, Chicago, Illinois; 8 Division of Infectious Diseases, Department of Pediatrics, Ann & Robert H. Lurie Children’s Hospital of Chicago, Chicago, Illinois; 9 Department of Pediatrics, Tulane University School of Medicine, New Orleans, Louisiana

## Abstract

**Importance:**

Since 1994, the US Department of Health and Human Services has published treatment guidelines for pregnant women living with HIV. Understanding how well prescribing patterns correspond with treatment guidelines could inform health policy and influence future clinical practice.

**Objectives:**

To compare antiretroviral prescribing practices over time among pregnant women living with HIV with Department of Health and Human Services treatment guidelines and identify factors associated with receiving recommended regimens.

**Design, Setting, and Participants:**

A prospective cohort study of 1582 pregnant women living with HIV were enrolled in the Pediatric HIV/AIDS Cohort Study Surveillance Monitoring of ART (antiretroviral therapy) Toxicities study between January 1, 2008, and June 30, 2017. The study was conducted at 18 academic research hospitals in the United States.

**Exposures:**

Antiretroviral medications (ARVs) prescribed during pregnancy.

**Main Outcomes and Measures:**

Proportion of regimens prescribed to pregnant women living with HIV qualifying as preferred or alternative according to Department of Health and Human Services guidelines, stratified by timing of initiation.

**Results:**

Of 1867 pregnancies (among 1582 pregnant women living with HIV with a mean [SD] age of 28.6 [6.1] years at conception), 1264 (67.7%) occurred among women self-identified as black, 480 (25.7%) self-identified as white, and 123 (6.6%) self-identified as other or unreported race/ethnicity. Antiretroviral medications were initiated prior to conception for 790 women (42.3%), resumed during pregnancy for 625 women (33.5%), and initiated during pregnancy for 452 women (24.2%). Only 925 pregnancies (49.5%) were associated with prescribed ARVs designated as preferred or alternative, while 492 (26.4%) involved ARVs with insufficient evidence for use during pregnancy and 136 (7.3%) involved ARVs that were not recommended during pregnancy. A higher proportion of treatment-naive pregnant women initiating ARVs were prescribed preferred or alternative ARVs compared with those resuming ARVs or those treated with ARVs before conception (316 of 452 [69.9%] vs 325 of 625 [52.0%] vs 284 of 790 [35.9%]; *P* < .001). A total of 91 of 452 women (20.1%) initiating ARVs during pregnancy were prescribed ARVs with insufficient evidence for use during pregnancy or not recommended during pregnancy. Among women resuming ARVs, those with a viral load greater than 1000 copies/mL early in pregnancy had higher odds of being prescribed guideline-recommended ARVs (adjusted odds ratio, 2.03 [95% CI, 1.33-3.10]) compared with those with a viral load of 400 copies/mL or less.

**Conclusions and Relevance:**

This study suggests that US ARV prescribing practices for pregnant women living with HIV do not align well with national guidelines. This finding is particularly concerning when treatment is initiated during pregnancy. Further research is needed to understand disparities between prescribing practices and evidence-based guideline recommendations.

## Key Points

**Question:**

Do antiretroviral prescribing patterns in the United States reflect Department of Health and Human Services prescribing guidelines for pregnant women living with HIV?

**Findings:**

In this cohort study of antiretroviral prescribing practices during 1867 pregnancies among women living with HIV, only 49.5% of antiretroviral prescriptions were classified as preferred or alternative according to Department of Health and Human Services guidelines.

**Meaning:**

More than half of the pregnant women living with HIV studied were prescribed antiretroviral regimens categorized by Department of Health and Human Services guidelines as having insufficient evidence for use in pregnancy or for which evidence indicates that use is not recommended.

## Teaser

This cohort study compares antiretroviral prescribing practices over time among pregnant women living with HIV with US Department of Health and Human Services treatment guidelines.

## Introduction

Since 1994, the US Department of Health and Human Services Panel on Treatment of HIV-Infected Pregnant Women and Prevention of Perinatal Transmission has published guidelines (Perinatal HIV Treatment Guidelines) for the use of antiretroviral medications (ARVs) during pregnancy. These guidelines are routinely amended to reflect the latest results of preclinical trials, clinical trials, and observational studies performed in the United States and globally, with expert opinion incorporated when evidence-based data are lacking. From their inception, the guidelines have promoted the importance of health care professionals sharing guideline recommendations with pregnant women living with HIV or those considering conception, to allow women to make informed choices about prophylaxis or treatment based on the latest efficacy and safety data.

In 1994, the guidelines^1^ solely addressed the use of zidovudine monotherapy to reduce the risk of infant HIV acquisition based on results of the landmark AIDS Clinical Trials Group Protocol 076.^2^ The 1998 Perinatal HIV Treatment Guidelines recognized that the benefits of ARV use among pregnant women living with HIV should be weighed against the risk of short-term and long-term adverse events for a woman and her developing fetus or newborn.^3^ In 2008, these guidelines were adapted to recommend use of triple ARVs for all pregnant women living with HIV, regardless of HIV disease status, and with lifetime continuation.^4^ The guidelines categorized combination ARV regimens and individual ARVs for use during pregnancy using terminology of *preferred*, *alternative*, *special circumstances*, *lacking sufficient evidence*, or *not recommended for use in pregnancy*, based on existing efficacy and safety data. Although these categories are informative, since 2011, the guidelines have advocated that women living with HIV and conceiving while being treated with fully suppressive regimens should continue using those regimens, with few exceptions.^5^ This provision recognized that ARV prescribing patterns may differ from guideline recommendations based on the timing of ARV treatment (ART) initiation, either prior to conception or during pregnancy.

To our knowledge, few studies have analyzed ARV prescribing practices during pregnancy. Griner and colleagues^6^ assessed temporal trends and maternal characteristics associated with ARV use during pregnancy between 1995 and 2009 using data from the Pediatric HIV/AIDS Cohort Study Surveillance Monitoring of ART Toxicities (SMARTT) study, concluding that many of the observed changes in ARV prescribing practices mirrored changes advocated in the evolving Perinatal HIV Treatment Guidelines. Phiri and colleagues,^7^ using Medicaid data, noted regional differences in ARV prescribing practices during pregnancy among women living with HIV.

To our knowledge, no study has quantitatively compared ARV prescribing practices during pregnancy with applicable Perinatal HIV Treatment Guidelines or evaluated maternal factors associated with prescribing preferred or alternative ARV regimens during pregnancy. Understanding how well prescribing practices align with guideline recommendations, as well as why deviations occur, is of great public health importance. Using the SMARTT cohort, our objectives were to assess how well ARV prescribing practices for pregnant women living with HIV reflected the Perinatal HIV Treatment Guidelines recommendations over time; to explore factors associated with clinician prescribing of ARV regimens during pregnancy designated by the guidelines as preferred or alternative; and to quantify regimen modifications or intensifications during pregnancy.

## Methods

### Study Population

**Figure 1.**
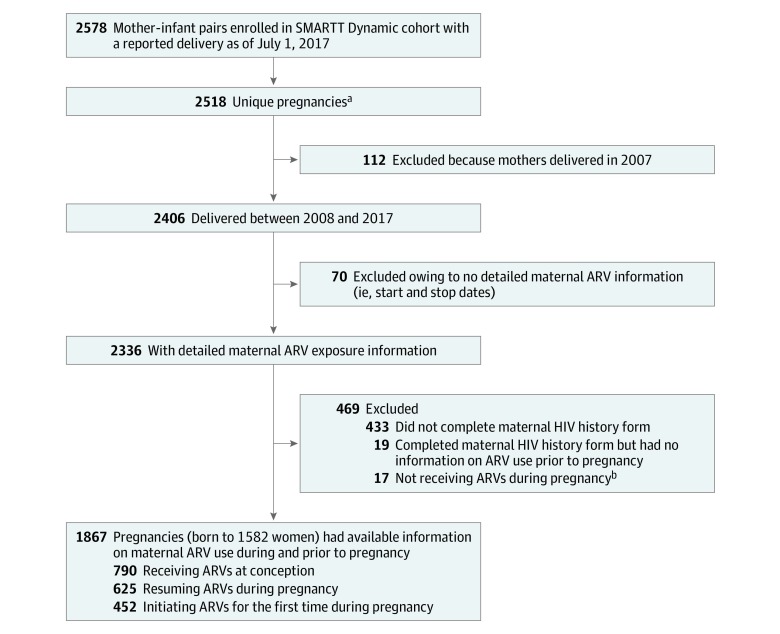
Flowchart of Study Population Selection ARV indicates antiretroviral medication; SMARTT, Surveillance Monitoring of ART Toxicities. ^a^Only unique pregnancies were included in the analysis (ie, gestations with twins or triplets were counted as 1 pregnancy). All repeated pregnancies of the same woman were included in the analysis because the maternal ARV regimens during pregnancy may change over time. ^b^Among the 17 pregnancies, 11 women received ARVs prior to pregnancy with 5 women also receiving ARVs during labor and delivery; 6 women did not receive ARVs prior to pregnancy, with 5 of these women receiving ARVs during labor and delivery .

Women enrolled in the SMARTT Dynamic cohort with a delivery between January 1, 2008, and June 30, 2017, were eligible for inclusion in this analysis, which was performed from June 1, 2017, to March 21, 2019. The SMARTT study includes 18 clinical research sites in the United States, as previously described.^8,9^ Since 2007, the SMARTT Dynamic cohort has been enrolling women living with HIV during pregnancy or at delivery and their infants and has followed up with them longitudinally after delivery. Antiretrovirals prescribed during pregnancy, including dates of initiation, are abstracted from each woman’s medical record. To quantify the proportion of prescribed ARVs corresponding to the Perinatal HIV Treatment Guidelines over time, only data from pregnancies with detailed ARV prescribing information during pregnancy and with available data about ARV use prior to conception were evaluated because guideline recommendations vary based on timing of ARV initiation. Antiretrovirals were not prescribed for 17 of 2336 pregnancies (0.7%; Figure 1); for 10 of these pregnancies, the women were treated with ARVs while in labor. We opted to exclude this small number of pregnancies because we could not confirm that these women were engaged in prenatal HIV care, making ARV prescribing decisions irrelevant. Three groups of women living with HIV were considered: those receiving ARVs at the time of conception, those previously treated with but not receiving ARVs at conception and who resumed ARVs during pregnancy, and treatment-naive women who started ARVs after conception. To evaluate the factors associated with being prescribed a guideline-designated preferred or alternative regimen, only women who resumed or initiated ARVs for the first time during pregnancy were considered. The institutional review board at each SMARTT site (Ann & Robert H. Lurie Children’s Hospital of Chicago; Baylor College of Medicine; Bronx Lebanon Hospital Center; Children’s Diagnostic & Treatment Center; New York University School of Medicine; Rutgers–New Jersey Medical School; St Jude Children’s Research Hospital; San Juan Hospital/Department of Pediatrics; SUNY Downstate Medical Center; Tulane University School of Medicine; University of Alabama at Birmingham; University of California, San Diego; University of Colorado, Denver; University of Florida, Center for HIV/AIDS Research, Education and Service; University of Illinois, Chicago; University of Miami; Keck Medicine of the University of Southern California; and University of Puerto Rico School of Medicine, Medical Science Campus) and at the Harvard T.H. Chan School of Public Health approved the protocol. All women provided written informed consent for study participation. This study followed the Strengthening the Reporting of Observational Studies in Epidemiology (STROBE) reporting guideline.

### Outcome Measures

We reviewed Perinatal HIV Treatment Guidelines from 2006 through 2016,^10^ creating hierarchical categorizations of ARV regimens and individual ARVs (eTable 1 and eTable 2 in the Supplement). These hierarchical categorizations included, from highest to lowest ranking, preferred, alternative, special circumstances, not mentioned (prescribing of an ARV or regimen during pregnancy not categorized in the guidelines), insufficient evidence for use during pregnancy (hereafter referred to as insufficient evidence), and not recommended. It was assumed that prescribing practices lagged 90 days from updated guideline issuance. For each ARV regimen, the categorization was defined first based on the prescribed ARV regimen (eTable 1 in the Supplement). If regimen evaluation resulted in a categorization higher than not recommended, further categorization was assigned based on individual ARVs in the regimen (eTable 2 in the Supplement). The lowest category assigned to a regimen or individual ARVs in a regimen was retained as the final categorization. We considered the first regimen prescribed during pregnancy, at conception, or thereafter, as reflecting the clinician’s initial prescribing decision. For women initiating or resuming ART after conception, prescribing practices were dichotomized by categories as preferred or alternative (together hereafter referred to as guideline recommended) compared with categories of not mentioned, insufficient evidence, and not recommended, excluding regimens qualifying as special circumstances, to identify factors associated with receiving guideline-recommended regimens. We opted to exclude pregnant women living with HIV who conceived while receiving an ART regimen from this analysis, as we did not have viral load (VL) information at the time of conception, because the guidelines were clear that if a woman’s VL was suppressed around the time of conception, with few exceptions, the regimen should not be changed, even if it was not a preferred or alternative regimen.

We also evaluated ART modification during pregnancy, defined as replacing 1 or more ARVs in a triple-ART regimen consisting of 2 ARV drug classes, and regimen intensification, defined as a change from a triple-drug regimen including 2 drug classes to a regimen including 4 or more ARVs or including drugs from 3 or more ARV classes, or introduction of an integrase strand transfer inhibitor as a replacement for a non–integrase strand transfer inhibitor ARV in a triple regimen.

### Potential Factors Associated With ARV Prescribing Patterns

Factors associated with receiving a guideline-recommended regimen were evaluated only among women resuming or initiating ARVs during pregnancy. We considered geographical region (Northeast, South, Midwest, West, or Puerto Rico), maternal characteristics (age at conception, highest educational level, household income at study enrollment, and race/ethnicity), pregnancy characteristics and comorbidities (trimester of prenatal care initiation, treatment for tuberculosis or psychiatric disorders in pregnancy, and presence of hepatitis B and/or C infection), and HIV-related characteristics (perinatal [acquired HIV at birth] or nonperinatal [acquired HIV later in life] mode of HIV acquisition, timing of HIV diagnosis, earliest HIV VL and CD4 cell count during pregnancy, and trimester of ARV initiation or resumption). We also considered maternal substance use based on self-reported use of alcohol, tobacco, or illicit drugs at or during the trimester prior to ARV initiation or resumption. As a proxy of maternal health insurance, we considered their infants’ Medicaid coverage vs any other insurance coverage including private insurance or no insurance.

### Statistical Analysis

We compared characteristics of eligible women in the SMARTT Dynamic cohort by timing of ARV initiation using χ^2^ tests and 1-way analysis of variance, as appropriate. The proportion of prescribed ARVs by guideline-designated categories was calculated and summarized by Department of Health and Human Services guideline index year, stratified by timing of ARV initiation.

Among women resuming or initiating ARVs during pregnancy, univariable logistic regression models were fit to estimate the unadjusted association of each covariate with receiving a guideline-recommended regimen (preferred or alternative). A multivariable model was then fit that included all covariates with *P* < .20 in univariable analysis and was reduced using a backward model selection approach, retaining only covariates with *P* < .10. The earliest maternal VL during pregnancy was retained as a covariate in the final multivariable model a priori because we hypothesized that VL would be associated with prescribing practices. Unadjusted and adjusted analyses were stratified by timing of ARV initiation, either newly initiated or resumed during pregnancy. For covariates with more than 5% missing values, indicators for missing data were created and included in analyses.

The prevalence of triple (3-drug, 2-class) ARV regimen modifications and intensifications were calculated among women prescribed ART during pregnancy, stratified by timing of initiation. Analyses were performed with SAS, version 9.4 (SAS Institute Inc). All *P* values were from 2-sided tests, and results were deemed statistically significant at *P* < .05.

## Results

Among 2003 women enrolled in the SMARTT Dynamic cohort who delivered between 2008 and 2017, 1582 women with 1867 pregnancies had ARV prescription information, including timing of ARV initiation (Figure 1). Of these 1867 pregnancies, 790 (42.3%) involved ARVs prescribed prior to conception, 625 (33.5%) involved resumption of ARVs during pregnancy, and 452 (24.2%) involved ARVs initiated for the first time during pregnancy. Overall, 1264 pregnancies (67.7%) occurred among women self-identified as black, 480 (25.7%) self-identified as white, 123 (6.7%) self-identified as other or unreported race/ethnicity, and 530 (28.4%) self-identified as Hispanic (Table 1).

**Table 1.** Maternal Characteristics of Pregnancies by Timing of ARV Initiation^a^

Characteristic	Pregnancies, No. (%)			
	Total (N = 1867)	Receiving ARVs at Conception (n = 790)	Resuming ARVs During Pregnancy (n = 625)	Initiating ARVs During Pregnancy (n = 452)
Race/ethnicity^b^				
Black	1264 (67.7)	512 (64.8)	443 (70.9)	309 (68.4)
White	480 (25.7)	228 (28.9)	146 (23.4)	106 (23.5)
Other	19 (1.0)	6 (0.8)	7 (1.1)	6 (1.3)
Hispanic ethnicity	530 (28.4)	243 (30.8)	152 (24.3)	135 (29.9)
Year of delivery				
2008	106 (5.7)	40 (5.1)	38 (6.1)	28 (6.2)
2009	132 (7.1)	35 (4.4)	38 (6.1)	59 (13.1)
2010	201 (10.8)	72 (9.1)	66 (10.6)	63 (13.9)
2011	250 (13.4)	91 (11.5)	86 (13.8)	73 (16.2)
2012	214 (11.5)	89 (11.2)	71 (11.4)	54 (11.9)
2013	241 (12.9)	101 (12.8)	89 (14.2)	51 (11.3)
2014	223 (11.9)	102 (12.9)	72 (11.5)	49 (10.8)
2015	220 (11.8)	104 (13.2)	72 (11.5)	44 (9.7)
2016	213 (11.4)	120 (15.2)	68 (10.9)	25 (5.5)
2017	67 (3.6)	36 (4.6)	25 (4.0)	6 (1.3)
Household annual income, $				
>30 000	237 (12.7)	133 (16.8)	63 (10.1)	41 (9.1)
>20 000-30 000	186 (10.0)	86 (10.9)	73 (11.7)	27 (6.0)
≤20 000	1353 (72.5)	545 (69.0)	456 (73.0)	352 (77.9)
Unknown	91 (4.9)	26 (3.3)	33 (5.3)	32 (7.1)
Education less than high school	568 (30.4)	244 (30.9)	191 (30.6)	133 (29.4)
Site region				
Puerto Rico	139 (7.4)	58 (7.3)	34 (5.4)	47 (10.4)
West	359 (19.2)	182 (23.0)	97 (15.5)	80 (17.7)
South	801 (42.9)	287 (36.3)	307 (49.1)	207 (45.8)
Midwest	153 (8.2)	62 (7.8)	53 (8.5)	38 (8.4)
Northeast	415 (22.2)	201 (25.4)	134 (21.4)	80 (17.7)
Age at conception, mean (SD), y	28.6 (6.1)	29.7 (6.2)	28.1 (5.8)	27.2 (6.0)
Gestational age at entry to prenatal care, mean (SD), wk	12.4 (6.7)	10.7 (5.7)	12.8 (6.7)	14.7 (7.3)
Trimester at entry to prenatal care^b^				
First	1281 (68.6)	622 (78.7)	409 (65.4)	250 (55.3)
Second	519 (27.8)	157 (19.9)	194 (31.0)	168 (37.2)
Third	65 (3.5)	10 (1.3)	22 (3.5)	33 (7.3)
Substance use prior to ARV initiation in pregnancy^b^				
Tobacco	213 (11.4)	NA	132 (21.1)	81 (17.9)
Alcohol	98 (5.2)	NA	63 (10.1)	35 (7.7)
Illicit drugs	126 (6.7)	NA	80 (12.8)	46 (10.2)
Maternal infections^c^				
Hepatitis B infection^b^	33 (1.8)	13 (1.6)	11 (1.8)	9 (2.0)
Hepatitis C infection^b^	24 (1.3)	8 (1.0)	13 (2.1)	3 (0.7)
Using Medicaid to pay child’s medical bills^b^	1515 (81.1)	622 (78.7)	534 (85.4)	359 (79.4)
Maternal medication use during pregnancy^d^				
Tuberculosis medication^b^	2 (0.1)	1 (0.1)	0	1 (0.2)
Psychiatric medications^b^	228 (12.2)	119 (15.1)	77 (12.3)	32 (7.1)
Perinatal HIV acquisition^b^	199 (10.7)	111 (14.1)	85 (13.6)	3 (0.7)
Maternal HIV diagnosis during pregnancy	314 (16.8)	0	0	314 (69.5)
Earliest HIV disease measures during pregnancy				
Viral load, copies/mL^b^				
>1000	772 (41.3)	126 (15.9)	342 (54.7)	304 (67.3)
>400-1000	111 (5.9)	26 (3.3)	41 (6.6)	44 (9.7)
≤400	962 (51.5)	622 (78.7)	238 (38.1)	102 (22.6)
CD4 count, cells/μL^b^				
<200	227 (12.2)	72 (9.1)	106 (17.0)	49 (10.8)
200-349	346 (18.5)	107 (13.5)	139 (22.2)	100 (22.1)
≥350	1260 (67.5)	590 (74.7)	372 (59.5)	298 (65.9)
Most intense ARV regimen during pregnancy^d^^,^^e^				
≥3 ARV drug classes	145 (7.8)	87 (11.0)	45 (7.2)	13 (2.9)
Triple ARV regimen from 2 drug classes	1576 (84.4)	670 (84.8)	513 (82.1)	393 (86.9)
≥3 NRTIs	94 (5.0)	9 (1.1)	46 (7.4)	39 (8.6)
Other ARV regimen	49 (2.6)	24 (3.0)	19 (3.0)	6 (1.3)
2 NRTIs	1 (0.05)	0	0	1 (0.2)
Zidovudine alone	1 (0.05)	0	1 (0.2)	0
No ARV or taking ARV for <3 d	1 (0.05)	0	1 (0.2)	0
Trimester of initiating ARV during pregnancy				
First	1184 (63.4)	790 (100)	301 (48.2)	93 (20.6)
Second	570 (30.5)	0	280 (44.8)	290 (64.2)
Third	113 (6.1)	0	44 (7.0)	69 (15.3)

Abbreviations: ARV, antiretroviral medication; NA, not applicable; NRTIs, nucleoside reverse transcriptase inhibitors.

^a^ Unique pregnancies that had a reported delivery after 2007 and had detailed maternal ARV exposure information and completed maternal HIV history form; women who did not receive any ARVs during pregnancy were excluded.

^b^ Some characteristics not available for all participants and handled as missing, including race/ethnicity (n = 104 missing); trimester of entry into prenatal care (n = 2); substance use not available among women initiating ARVs prior to conception; tobacco use (n = 13); alcohol use (n = 11); illicit drug use (n = 15); hepatitis B status (n = 145); hepatitis C status (n = 484); using Medicaid to pay child’s medical bills (n = 10); tuberculosis medication taken in pregnancy (n = 11); psychiatric medication taken in pregnancy (n = 11); perinatal HIV acquisition (n = 46); maternal HIV diagnosis in pregnancy (n = 15); earliest viral load in pregnancy (n = 22); and earliest CD4 cell count in pregnancy (n = 34).

^c^ Hepatitis B infection was defined as having a positive hepatitis surface antigen test result. Hepatitis C was defined as having a positive hepatitis C RNA test result.

^d^ Tuberculosis or psychiatric medications taken during pregnancy with potential interactions with ARV drugs were included.

^e^ The most intense ARV regimen prescribed during pregnancy was defined based on duration of use during pregnancy of 3 or more days and a hierarchy from the most to least intense as (1) ARV regimens consisting of 3 or more ARVs, (2) triple ARV regimens from 2 drug classes, (3) 3 or more NRTIs, (4) other ARV regimens, (5) 2 NRTIs, (6) zidovudine with or without single-dose nevirapine, and (7) no ARVs. If a switch in prescribed regimens occurred during pregnancy, the most intense regimen was selected, so long as it was used for 3 or more days during pregnancy. Where a regimen switch occurred within the same intensification category, the regimen used for the longest duration was selected for analysis.

Some maternal characteristics differed by timing of ARV initiation (Table 1). Fewer women resuming ARVs during pregnancy were Hispanic compared with those conceiving while receiving ARVs or initiating ARVs during pregnancy. Women conceiving while receiving ARVs had a higher mean age at conception, an earlier engagement in antenatal care, and a higher proportion were virally suppressed (HIV VL ≤400 copies/mL) and had a CD4 cell count of 350 cells/μL or more at the earliest testing during pregnancy compared with those initiating or resuming ARVs in pregnancy.

**Figure 2.**
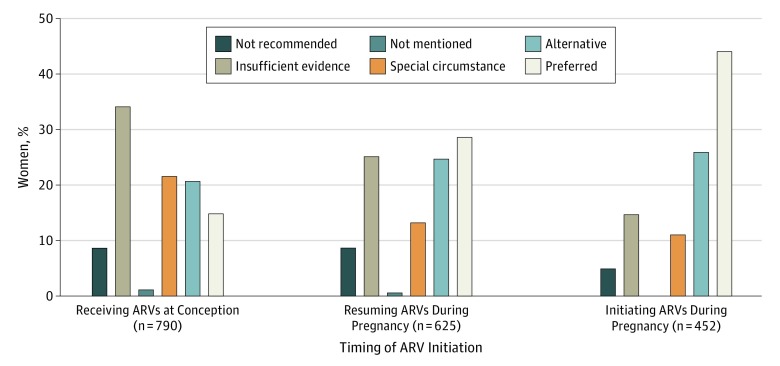
Antiretroviral (ARV) Prescribing Patterns by Timing of Initiation According to US Perinatal HIV Treatment Guidelines A total of 790 women received ARVs at conception, 625 resumed ARVs during pregnancy, and 452 initiated ARVs during pregnancy.

Only 925 of the 1867 pregnancies (49.5%) were associated with a prescribed ARV drug or regimen with a guideline designation of preferred or alternative, 297 (15.9%) were prescribed ARVs recommended for use under special circumstances, 492 (26.4%) were prescribed ARVs with insufficient evidence for use during pregnancy, 136 (7.3%) involved ARVs not recommended for use during pregnancy, and 11 (0.9%) were prescribed ARVs not mentioned in the guidelines (see the eFigure in the Supplement for prescribed ARVs over time). A higher proportion of pregnancies in which ARVs were initiated involved preferred or alternative ARVs than those in which ARVs were resumed or taken from conception (316 of 452 [69.9%] vs 325 of 625 [52.0%] vs 284 of 790 [35.9%]; *P* < .001) (Figure 2). Conversely, a higher proportion of women conceiving while receiving ARVs were prescribed ARVs designated as having insufficient evidence for use during pregnancy (268 of 790 [33.9%]) compared with 156 of 625 women (25.0%) resuming ARVs and 68 of 452 women (15.0%) newly initiating ARVs (*P* < .001). Women initiating ARVs during pregnancy were less often prescribed ARVs not recommended for use during pregnancy (23 of 452 [5.1%]) than those conceiving while receiving ARVs (63 of 790 [8.0%]) or resuming ARVs during pregnancy (50 of 625 [8.0%]). Antiretroviral prescribing practices by the timing of ARV initiation during pregnancy, when compared with the guidelines, showed similar trends in each guideline index year to overall trends (eFigure in the Supplement). We evaluated ART regimens prescribed to treatment-naive women living with HIV prior to 2015 for which the guidelines found insufficient evidence for use during pregnancy and found that 55 of 63 prescribed ARV regimens (87.3%) would have been classified as preferred or alternative options in subsequent guideline releases.

Among 543 pregnancies with ARV resumption, women with their earliest VL greater than 1000 copies/mL had more than 2-fold greater odds of being prescribed a guideline-recommended regimen (adjusted odds ratio [aOR], 2.03 [95% CI, 1.33-3.10]) compared with those with a VL of 400 copies/mL or less (Table 2). Conversely, women with an earliest CD4 cell count less than 200 cells/μL during pregnancy had decreased odds of being prescribed a guideline-recommended regimen (aOR, 0.52 [95% CI, 0.30-0.88]) compared with those with a CD4 cell count of 350 cells/μL or more. Women with unknown hepatitis B infection status during pregnancy had lower odds of being prescribed a guideline-recommended regimen compared with those without hepatitis B coinfection (aOR, 0.36 [95% CI, 0.16-2.25]), and those prescribed ARVs under 2007-2013 guidelines had increased odds of being prescribed recommended regimens than those prescribed ARVs between 2014 and 2015 (aOR, 2.63 [95% CI, 1.78-3.89]).

**Table 2.** Adjusted ORs of Being Prescribed Preferred or Alternative Antiretroviral Regimens on Resumption of Treatment During Pregnancy Among 543 Pregnancies

Covariate	Adjusted OR (95% CI)^a^
Earliest CD4 count, cells/μL	
≥350	1 [Reference]
200-349	0.56 (0.35-0.89)
<200	0.52 (0.30-0.88)
Earliest viral load, copies/mL	
≤400	1 [Reference]
>400-1000	1.33 (0.63-2.79)
>1000	2.03 (1.33-3.10)
Hepatitis B infection	
No	1 [Reference]
Unknown	0.36 (0.16-0.80)
Yes	0.60 (0.16-2.25)
Index year of DHHS guideline	
2014-2015	1 [Reference]
2007-2013	2.63 (1.78-3.89)
2006	1.26 (0.48-3.31)

Abbreviations: DHHS, US Department of Health and Human Services; OR, odds ratio.

^a^ Adjusted OR of being prescribed preferred or alternative antiretroviral regimens by participant covariate compared with the reference group of prescribed antiretroviral not recommended, with insufficient evidence, or not mentioned in the guidelines, with 95% CI. Women who received antiretrovirals for special circumstances were excluded from analysis.

Among 403 women prescribed ARVs for the first time during pregnancy, the index year of the guidelines was the only covariate associated with prescribing practices after adjusting for earliest VL during pregnancy (Table 3). Compared with ARV prescriptions written for pregnant women living with HIV in the guideline index year 2014-2015, prescriptions for the guideline periods 2007 to 2013 had more than 2-fold greater odds of being prescribed a guideline-recommended regimen (aOR, 2.23 [95% CI, 1.31-3.80]).

**Table 3.** Adjusted ORs of Being Prescribed Preferred or Alternative Antiretroviral Regimens on First Initiation of Treatment During Pregnancy Among 403 Pregnancies

Covariate	Adjusted OR (95% CI)^a^
Index year of DHHS guideline	
2014-2015	1 [Reference]
2007-2013	2.23 (1.31-3.80)
2006	4.22 (0.50-35.46)
Earliest viral load, copies/mL	
≤400	1 [Reference]
>400-1000	2.42 (0.84-6.98)
>1000	1.27 (0.73-2.21)

Abbreviations: DHHS, US Department of Health and Human Services; OR, odds ratio.

^a^ Adjusted OR of being prescribed preferred or alternative antiretroviral regimens by participant covariate compared with the reference group of prescribed antiretrovirals not recommended, or with insufficient evidence, or not mentioned in the guidelines, with 95% CI. Women who received antiretrovirals for special circumstances were excluded from analysis.

Among 1867 pregnancies evaluated in this analysis, 1641 (87.9%) represented pregnancies in which ART consisting of 3 drugs from 2 ARV drug classes was prescribed. Antiretroviral therapy modification occurred in 230 (14.0%) of these pregnancies. A higher proportion of regimen modifications occurred in pregnancies in which ART was prescribed before conception (134 of 707 [19.0%]) than when ART was resumed during pregnancy (64 of 530 [12.1%]) or newly initiated during pregnancy (32 of 404 [7.9%]) (*P* < .001). Antiretroviral therapy intensification occurred in 157 of 1641 pregnancies (9.6%) overall, but again, it occurred more often for women conceiving while receiving ART (85 of 707 [12.0%]) compared with those resuming ART during pregnancy (48 of 530 [9.1%]) or initiating ART during pregnancy (24 of 404 [5.9%]) (*P* = .02).

## Discussion

In what is to our knowledge the first analysis of US clinicians’ ARV prescribing practices compared with recommendations of the US Department of Health and Human Services Perinatal HIV Treatment Guidelines, we found that only 69.9% of women living with HIV initiating ARVs in pregnancy were prescribed a guideline-designated preferred or alternative regimen and 20.1% were prescribed regimens not recommended during pregnancy or for which there was insufficient evidence for use during pregnancy. A lower proportion of women living with HIV resuming ARVs during pregnancy (52.0%) or when receiving ARVs at conception (35.9%) were prescribed ARVs designated by the guidelines as preferred or alternative. Although these results could indicate a high rate of clinician noncompliance with the guidelines, they could equally reflect compliance with other guidelines directives. For example, the guidelines recommend that a suppressive regimen at conception should not be changed, with few exceptions.^11^ In addition, guidelines encourage consideration of genotypic drug resistance testing, unacceptable adverse effects of prior ARVs, comorbidities and associated drug interactions, and dosing convenience when prescribing ART during pregnancy, while promoting prescriber flexibility to accommodate a woman’s individual circumstances and preferences.^12^

In this cohort, 26.4% of pregnancies involved prescribed ARVs with a guideline classification of insufficient evidence for use during pregnancy. This occurred most frequently when ARVs were prescribed before conception (33.9%) and may well have reflected guideline recommendations that indicated, with few exceptions, that women receiving a suppressive regimen prior to conception should continue receiving that regimen throughout pregnancy. However, 15.0% of treatment-naive pregnant women initiated ARVs with insufficient safety and efficacy data. This practice can pose a risk of lower ARV drug levels during pregnancy and/or may be associated with adverse fetal outcomes. Primary transmitted ARV drug resistance may have limited ARV prescribing options. Resistance rates range from 6% to 16% among adults with HIV in the United States, and in a small cohort of pregnant women living with HIV, the rates of resistance during pregnancy were 55% among those with perinatally acquired HIV and 17% among those with nonperinatally acquired HIV.^13,14,15,16^ However, resistance results are not immediately available in most clinic settings, and the 2016 guidelines were updated to recommend immediate initiation of ART for treatment-naive pregnant women while awaiting resistance results.^17^ Prescriptions written in guideline periods prior to 2014 were more likely to align with guideline-preferred or alternative regimens. Starting in 2014, the decrease in the proportion of preferred or alternative regimens was associated primarily with prescribing of rilpivirine-containing regimens, dolutegravir-containing regimens, and cobicistat-boosted elvitegravir-containing regimens, all of which lacked sufficient evidence for use during pregnancy at that time. These same ARVs were associated with the decrease in the proportion of preferred and alternative ARVs prescribed in the 2015 guideline period.

In cases in which prescriptions written prior to the 2015 guidelines for treatment-naive women were categorized as having insufficient evidence for use during pregnancy, 87.3% were subsequently reclassified as preferred or alternative in later guidelines. These ARVs included rilpivirine, ritonavir-boosted darunavir, tenofovir disoproxil fumarate, and ritonavir-boosted atazanavir, listed by most to least frequently prescribed, all of which represented appropriate ARVs for treatment of nonpregnant adults living with HIV. However, data providing definitive evidence of safety for the pregnancy outcome and the exposed fetus were insufficient at the time. Consistent use of reporting mechanisms such as the Antiretroviral Pregnancy Registry^18^ by prescribers may decrease lag time between availability of new ARVs and informative pregnancy safety and efficacy data. Although we did not collect data on the reasons for therapy modifications or intensifications, it is likely that most changes were due to unacceptable adverse effects or failure to achieve VL suppression. The 2007 guidelines first addressed persistent viremia despite adequate treatment, stressing the importance of selecting potent, well-tolerated regimens during pregnancy to achieve viral suppression. An Italian study found that ARV modification among 662 pregnant women living with HIV was independently associated with a 66% greater risk of unsuppressed delivery VLs.^19^ In another observational cohort in France, 411 pregnant women living with HIV taking ART from conception who underwent regimen switching owing to safety concerns in the first trimester had similar rates of virologic failure compared with those whose regimen was switched for a nonsafety reason (19.3% vs 15.6%; adjusted hazard ratio, 1.0 [95% CI, 0.7-1.4]).^20^ Given the high frequency of regimen modifications and intensifications at more than 20%, this may be an important area to address in future guideline updates.

### Limitations

Although detailed ARV data maintained on women participating in the SMARTT cohort is a key strength of this study and has enabled an in-depth comparison of prescribing practices with US guideline recommendations, our study is not without limitations. Notably, SMARTT sites are based primarily at academic institutions, and findings may not be generalizable to all US locations. Second, we did not have access to data that would have prompted clinicians to prescribe nonpreferred or alternative regimens, such as findings of ARV drug resistance, prior ARV adverse events, existence of high third-trimester VL, or the expressed preferences of pregnant women living with HIV. Third, we assumed it would take up to 3 months after guideline release before clinician prescribing practices changed. The SMARTT study sites represent academic institutions, which may disproportionately adopt revised guidelines more rapidly. However, the fact that none of the prescriptions for the 63 ART regimens categorized as insufficient evidence for use during pregnancy among treatment-naive women were prescribed within 90 days of updated guidelines, reflecting early adoption of newly released guidelines, suggests that identified variances between prescribing practices and guidelines were not associated with the 3-month lag that we selected for analysis purposes. Furthermore, we anticipate that a longer lag time would have had little association with the observed results. We excluded 433 pregnancies for which data on use of ARVs prior to the current pregnancy were not available, precluding differentiation between women resuming ARVs during pregnancy and women newly initiating ARVs. We speculate that since 389 (89.8%) of these pregnancies occurred prior to 2010, when the SMARTT study began collecting information on use of ARVs prior to the current pregnancy, a higher proportion of these women were likely prescribed a preferred or alternative regimen because the earlier guideline periods were associated with prescribing patterns more closely aligned with guideline recommendations.

## Conclusions

Among pregnant women living with HIV, disease control through the use of ART must be balanced with its efficacy in preventing perinatal HIV transmission and its safety for both the woman and the fetus. The US Perinatal ARV Treatment guidelines are continually updated to reflect recent evidence-based safety and efficacy outcomes. Our study demonstrates discordance between guideline recommendations and actual prescribing practices, even when prescribing for treatment-naive pregnant women living with HIV. This finding may reflect the dilemma that pregnant women are typically excluded from clinical trials investigating the safety and tolerability of new ARV medications in an effort to protect the fetus, introducing increased lag time between US Food and Drug Administration approval of new drugs and data on which revisions to the guidelines for pregnant women living with HIV are justified. At the same time, our findings highlight the importance of conducting quantitative and qualitative research to better understand the extent to which individual, social, institutional, and structural determinants are associated with prescribing practices that do not align with guideline recommendations.

## Notes

Supplement. **eTable 1.** US Perinatal HIV Treatment Guidelines Classification of ARV Regimens **eTable 2.** US Perinatal HIV Treatment Guidelines Classification of Individual ARVs **eFigure.** Longitudinal Prescribing Patterns According to Perinatal HIV Treatment Guideline Categories by Timing of ARV Initiation

## References

[1] Centers for Disease Control and Prevention Recommendations of the US Public Health Service Task Force on the use of zidovudine to reduce perinatal transmission of human immunodeficiency virus. MMWR Recomm Rep. 1994;43(RR-11):-. 7913986

[2] Connor EM, Sperling RS, Gelber R, Reduction of maternal–infant transmission of human immunodeficiency virus type 1 with zidovudine treatment: Pediatric AIDS Clinical Trials Group Protocol 076 Study Group. N Engl J Med. 1994;331(18):1173-1180. doi:10.1056/NEJM199411033311801 7935654

[3] Centers for Disease Control and Prevention Public Health Service Task Force recommendations for the use of antiretroviral drugs in pregnant women infected with HIV-1 for maternal health and for reducing perinatal HIV-1 transmission in the United States. MMWR Recomm Rep. 1998;47(RR-2):1-30. 9461044

[4] Public Health Service Task Force Recommendations for use of antiretroviral drugs in pregnant HIV-infected women for maternal health and interventions to reduce perinatal HIV transmission in the United States. https://aidsinfo.nih.gov/contentfiles/PerinatalGL001020.pdf. Published July 8, 2008. Accessed December 2, 2018.

[5] Centers for Disease Control and Prevention Recommendations for use of antiretroviral drugs in pregnant HIV-1–infected women for maternal health and interventions to reduce perinatal HIV transmission in the United States. https://npin.cdc.gov/publication/recommendations-use-antiretroviral-drugs-pregnant-hiv-1-infected-women-maternal-health. Accessed February 6, 2019. 12489844

[6] Griner R, Williams PL, Read JS, ; Pediatric HIV/AIDS Cohort Study In utero and postnatal exposure to antiretrovirals among HIV-exposed but uninfected children in the United States. AIDS Patient Care STDS. 2011;25(7):385-394. doi:10.1089/apc.2011.0068 21992592 PMC3125552

[7] Phiri K, Fischer MA, Mogun H, Trends in antiretroviral drug use during pregnancy among HIV-infected women on Medicaid: 2000-2007. AIDS Patient Care STDS. 2014;28(2):56-65. doi:10.1089/apc.2013.0165 24517538 PMC3926172

[8] Van Dyke RB, Chadwick EG, Hazra R, Williams PL, Seage GR III The PHACS SMARTT Study: assessment of the safety of in utero exposure to antiretroviral drugs. Front Immunol. 2016;7:199. doi:10.3389/fimmu.2016.00199 27242802 PMC4876360

[9] Williams PL, Hazra R, Van Dyke RB, ; Pediatric HIV/AIDS Cohort Study Antiretroviral exposure during pregnancy and adverse outcomes in HIV-exposed uninfected infants and children using a trigger-based design. AIDS. 2016;30(1):133-144. 26731758 10.1097/QAD.0000000000000916 PMC4704129

[10] US Department of Health and Human Services Archived perinatal guidelines. https://aidsinfo.nih.gov/guidelines/archive/perinatal-guidelines. Accessed December 12, 2018.

[11] Perinatal HIV Guidelines Working Group Recommendations for use of antiretroviral drugs in pregnant HIV-infected women for maternal health and interventions to reduce perinatal HIV transmission in the United States. 2007 https://aidsinfo.nih.gov/ContentFiles/PerinatalGL02112007901.pdf. Accessed November 30, 2016.

[12] Panel on Treatment of HIV-Infected Pregnant Women and Prevention of Perinatal Transmission Recommendations for use of antiretroviral drugs in pregnant HIV-1–infected women for maternal health and interventions to reduce perinatal HIV transmission in the United States. https://aidsinfo.nih.gov/contentfiles/PerinatalGL003482.pdf. Accessed February 6, 2019.

[13] Wheeler WH, Ziebell RA, Zabina H, ; Variant, Atypical, and Resistant HIV Surveillance Group Prevalence of transmitted drug resistance associated mutations and HIV-1 subtypes in new HIV-1 diagnoses, U.S.—2006. AIDS. 2010;24(8):1203-1212. doi:10.1097/QAD.0b013e3283388742 20395786

[14] Weinstock HS, Zaidi I, Heneine W, The epidemiology of antiretroviral drug resistance among drug-naive HIV-1–infected persons in 10 US cities. J Infect Dis. 2004;189(12):2174-2180. doi:10.1086/420789 15181563

[15] Ross L, Lim ML, Liao Q, Prevalence of antiretroviral drug resistance and resistance-associated mutations in antiretroviral therapy-naïve HIV-infected individuals from 40 United States cities. HIV Clin Trials. 2007;8(1):1-8. doi:10.1310/hct0801-1 17434843

[16] Lazenby GB, Mmeje O, Fisher BM, Antiretroviral resistance and pregnancy characteristics of women with perinatal and nonperinatal HIV infection. Infect Dis Obstet Gynecol. 2016;2016:4897501. doi:10.1155/2016/4897501 27413359 PMC4930810

[17] Panel on Treatment of HIV-Infected Pregnant Women and Prevention of Perinatal Transmission Recommendations for use of antiretroviral drugs in pregnant HIV-1–infected women for maternal health and interventions to reduce perinatal HIV transmission in the United States. 2016 https://aidsinfo.nih.gov/contentfiles/PerinatalGL003422.pdf. Accessed November 5, 2016.

[18] Antiretroviral Pregnancy Registry Steering Committee The antiretroviral pregnancy registry interim report: 1 January 1989 through 31 January 2019. http://www.apregistry.com/forms/interim_report.pdf. Published June 2019. Accessed February 6, 2019.

[19] Floridia M, Ravizza M, Pinnetti C, ; Italian Group on Surveillance on Antiretroviral Treatment in Pregnancy Treatment change in pregnancy is a significant risk factor for detectable HIV-1 RNA in plasma at end of pregnancy. HIV Clin Trials. 2010;11(6):303-311. doi:10.1310/hct1106-303 21239358

[20] Peyronnet V, Warszawski J, Sibiude J, Does changing antiretroviral therapy in the first trimester of pregnancy for safety concerns have an impact on viral suppression? J Acquir Immune Defic Syndr. 2019;80(5):574-584. doi:10.1097/QAI.0000000000001954 30649033

