# Supplementary material for: Antiretroviral Prescribing Practices Among Pregnant Women Living With HIV in the United States, 2008-2017
Source: JAMA Netw Open. 2019 Dec 18;2(12):e1917669. doi: 10.1001/jamanetworkopen.2019.17669 (PMC6991210; doi:10.1001/jamanetworkopen.2019.17669)
Supplement: Supplement. — eTable 1. US Perinatal HIV Treatment Guidelines Classification of ARV Regimens eTable 2. US Perinatal HIV Treatment Guidelines Classification of Individual ARVs eFigure. Longitudinal Prescribing Patterns According to Perinatal HIV Treatment Guideline Categories by Timing of ARV Initiation [file jamanetwopen-2-e1917669-s001.pdf]

Supplementary Online Content

Powis KM, Huo Y, Williams PL, et al; Pediatric HIV/AIDS Cohort Study (PHACS). Antiretroviral prescribing practices among pregnant women living with HIV in the United States, 2008-2017. *JAMA Netw Open.* 2019;2(12):e1917669. doi:10.1001/jamanetworkopen.2019.17669

eTable 1. US Perinatal HIV Treatment Guidelines Classification of ARV Regimens
eTable 2. US Perinatal HIV Treatment Guidelines Classification of Individual ARVs
eFigure. Longitudinal Prescribing Patterns According to Perinatal HIV Treatment Guideline Categories by Timing of ARV Initiation

This supplementary material has been provided by the authors to give readers additional information about their work.

© 2019 Powis KM et al. *JAMA Network Open.*

eTable 1. US Perinatal HIV Treatment Guidelines Classification of ARV Regimens

DHHS Index Year[a]	Preferred/alternative regimens	Not recommended/insufficient data regimens
2006	(none stated)	
2007	HAART – preferred[b]<br>3+ NRTIs – alternative[c]	▪ ddI+d4T-containing regimens – not recommended<br>▪ d4T+ZDV-containing regimens – not recommended<br>▪ Regimens containing ≤ 2 drugs – not recommended
2008-2009	(same as 2007)	▪ (Same as 2007)
2010	(same as 2007)	▪ (Same as 2007)
2011-2013	(same as 2007)	▪ (Same as 2007)
2014	HAART with one of the following dual NRTI backbones:<br>ABC+3TC<br>TDF+3TC<br>TDF+FTC<br>ZDV+3TC<br>COBI/EVG/TDF/FTC – preferred for treatment-experienced women	▪ ZDV+3TC+ABC – not recommended<br>▪ Any HAART regimen not containing one of preferred dual NRTI backbones – not recommended<br>▪ COBI/EVG/TDF/FTC – insufficient evidence for treatment-naïve women<br>▪ Regimens containing ≤ 2 drugs – not recommended
2015	(same as 2014)	▪ (Same as 2014)<br>▪ HAART regimen containing COBI boosted DRV or ATV is alternative regimen for treatment-experienced women and Table A2 COBI classifications should be analyzed accordingly.<br>▪ HAART regimen HAART regimen containing COBI boosted DRV or ATV is not recommended for treatment-naïve women
2016	HAART with one of the following dual NRTI backbones – recommended for ARV experienced:<br>ABC+3TC<br>TDF+3TC<br>TDF+FTC<br>ZDV+3TC<br>HAART with one of the following 3 dual NRTI backbones – recommended for ARV-naïve:<br>ABC+3TC<br>TDF+3TC<br>TDF+FTC<br>HAART with ZDV+3TC backbone – alternative in ARV-naïve<br>COBI/EVG/TDF/FTC – preferred for treatment-experienced women	▪ RPV/TAF/FTC (Odefsey) – insufficient data<br>▪ ZDV+3TC+ABC – not recommended<br>▪ Any HAART regimen not containing one of the 4 dual NRTI backbones – not recommended<br>▪ COBI/EVG/TDF/FTC (Stribild) – insufficient evidence for treatment-naïve women<br>▪ Regimens containing ≤ 2 drugs – not recommended<br>▪ HAART regimen containing COBI boosted DRV or ATV is an alternative regimen for treatment-experienced women and Table A2 COBI classifications should be analyzed accordingly.<br>▪ HAART regimen containing COBI boosted DRV or ATV is not recommended for treatment-naïve women

[a]DHHS index year was defined as from 90 days after the release of a guideline through 89 days after issuance of an updated DHHS Guideline.
[b]HAART: at least 3 drugs from at least 2 classes.
[c]Any woman receiving ≥3 NRTIs was assumed to have not required a more potent regimen for her own health.

© 2019 Powis KM et al. *JAMA Network Open.*

eTable 2. US Perinatal HIV Treatment Guidelines Classification of Individual ARVs

	2006	2007	2008	2009	2010	2011	2012	2014	2015	2016
**NRTIs**										
**ZDV**	**P**	P	P	P	P	P	P	P	P	P
**3TC**	**P**	P	P	P	P	P	P	P	P	P
**FTC**	**A**	A	A	A	A	A	A	P	P	P
**ABC**	**A**	A	A	A	A	A	A	P	P	P
**TDF**	**I**	I	I	S	S	A	A	P	P	P
**D4T**	**A**	A	A	A	A	A	S	N	N	N
**DDI**	**A**	A	A	A	A	A	S	N	N	N
**DDC**	**N**	N	N	NM	NM	NM	NM	NM	NM	NM
**TAF**	NM	NM	NM	NM	NM	NM	NM	NM	NM	I
**NNRTIs**										
**NVP**	**P**	P	P	P	P	P	P	A	N*/A	N
**EFV**	**N^{T}/N^{A}**	N	N	S	S	S	S	P after 8 wks	P after 8 wks	A
**DEL**	**N**	N	N	NM	NM	NM	NM	NM	NM	NM
**ETR**	**NM**	NM	NM	I	I	I	I	N*/A	N*/A	N*/A
**RPV**	**NM**	NM	NM	NM	NM	I	I	I	A	A
**PIs**										
**LPV/R**	**P**	P	P	P	P	P	P	P	A	A
**DRV/R**	I	I	I	I	I	I	S	A	P	P
**DRV**	I	N	N	N	N	N	N	N	N	N
**ATZ/R**	I	I	I	A	A	A	P	P	P	P
**ATZ**	I	I	I	N	N	N	N	N	N	N
**NFV**	**P**	N	A	A	A	S	S	N	N	N
**RTV**	**N^{A}**	**N^{A}**	**N^{A}**	**N^{A}**	**N^{A}**	**N^{A}**	**N^{A}**	**N^{A}**	**N^{A}**	**N^{A}**
**TPV/R**	I	I	I	I	I	I	I	N*/A	N*/A	N*/A
**TPV**	I	I	I	N	N	N	N	N*/A	N	N
**SAQ/R**	**A**	A	A	A	A	A	A	A	N	N
**SAQ**	**A**	N	N	N	N	N	N	N	N*/A	N
**IDV/R**	**A**	A	A	A	A	S	S	N	N	N
**IDV**	NM	N	N	N	N	N	N	N	N	N
**FPV/R**	I	I	I	I	I	I	I	I	I	I
**FPV**	I	I	I	N	N	N	N	N	N	N
**AMP**	I	I	I	NM	NM	NM	NM	NM	NM	NM
**COBI**	NM	NM	NM	NM	NM	NM	NM	N*/A	I	I*/A
**ENTRY INHIBITORS**										
**T-20**	I	I	I	I	I	I	I	N*/A	N*/A	N*/A
**MVC**	NM	I	I	I	I	I	I	I	I	I
**INTEGRASE INHIBITORS**										
**EVG**	NM	NM	NM	NM	NM	NM	NM	I	I	I
**RAL**	NM	I	I	I	I	I	S	A	P	P
**DTG**	NM	NM	NM	NM	NM	NM	NM	I	I	I

Legend: P = Preferred; A = Alternative; S = Special Circumstances; N = Not recommended; N^{A}=Not recommended if sole Protease Inhibitor (PI); if used to boost levels of another PI, rating based on other PI; N*/A = Not recommended in treatment naïve women but alternative regimen in treatment experienced; I = Insufficient Evidence; I*/A= Insufficient data to initiate in pregnancy among treatment naïve women but alternative for treatment experienced; NM = Not Mentioned.

© 2019 Powis KM et al. *JAMA Network Open.*

eFigure. Longitudinal Prescribing Patterns According to Perinatal HIV Treatment Guideline Categories by Timing of ARV Initiation

A. ARVs from Conception (N=790)

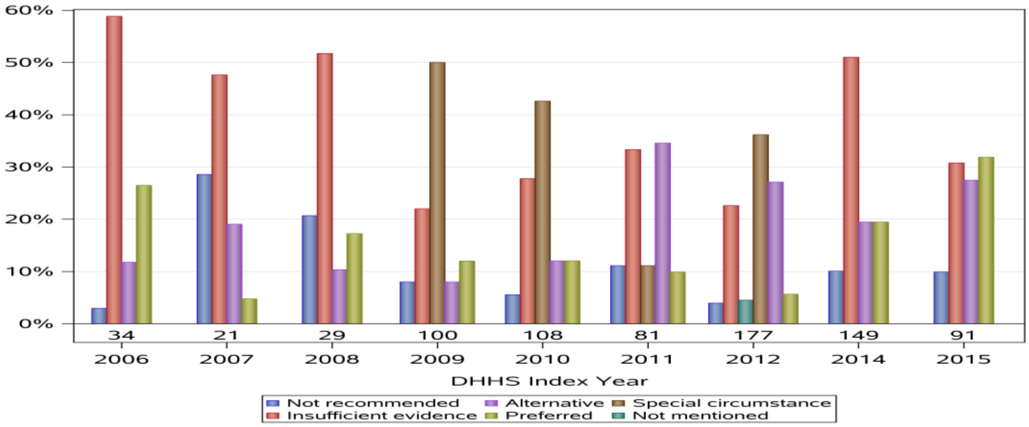

B. Resuming ARVs in Pregnancy (N=625)

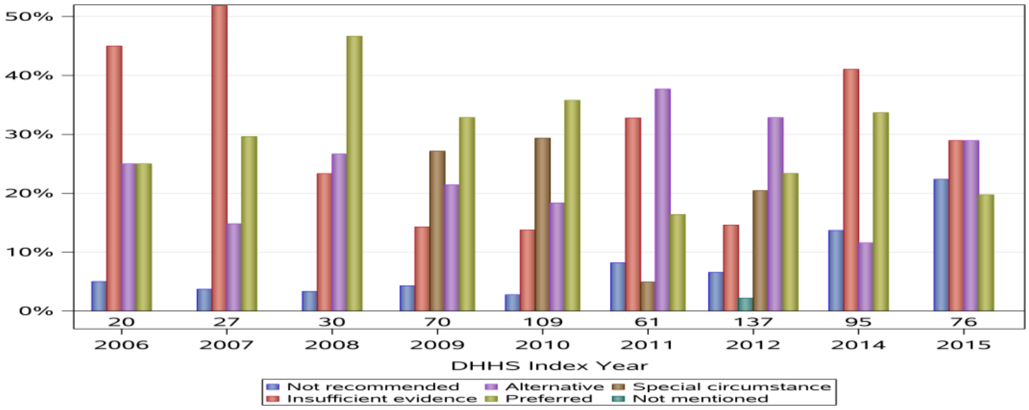

C. Initiating ARVs in Pregnancy (N=452)

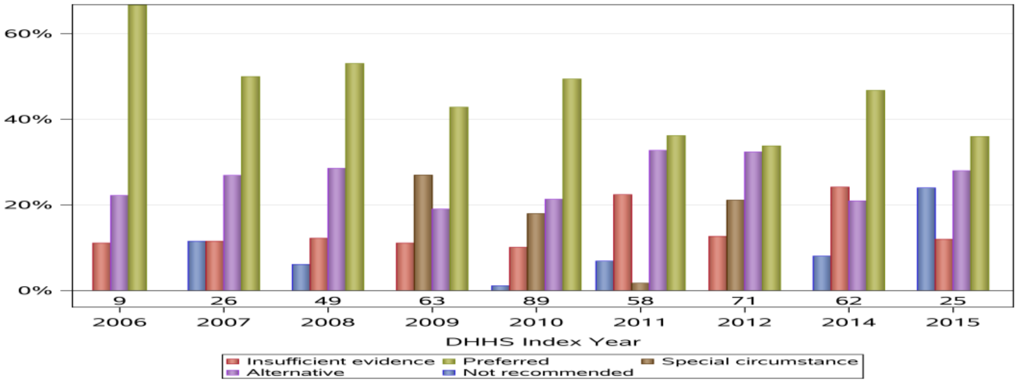

Number above the x-axis represents number of deliveries in the Guideline Index year.

Abbreviations: ARVs – antiretrovirals; DHHS – Department of Health and Human Services

© 2019 Powis KM et al. *JAMA Network Open.*
